# The General Infirmary, Leeds

**Published:** 1893-11-04

**Authors:** 


### Editor's Letter-Box.

[Our correspondents are reminded that proxility is a great bar to publication, and that brevity of style and conciseness of statement greatly facilitate early insertion.]

### THE GENERAL INFIRMARY, LEEDS.

Mr. Thomas Blair, General Manager, writes under date October 25th: "Referring to your article, 'Hospital Administration,' in The Hospital of October 21st, at page 45, permit me to point out an error under the heading 'Beds for Occupation.' In our annual report for 1892, on page 8, you will find an explanation on this point, viz., that during seven months we had 326 beds for occupation, and for the remaining five months 344, and not 436, as stated by you. On the same page of the report you will also find it explained that while the new extension increases the total accommodation to 436 beds, it is proposed to have only 394 made up for occupation. The new buildings were formally opened in 1892, but only a portion of them were then occupied; the 394 beds were completed and ready for occupation during the first quarter of the present year, so that this year, like last, will also be an unequal year—we shall have had 344 beds for two months, and 394 for ten months. As I cannot quite make out your percentages, I have sent you a copy of our balance-sheet marked 'Maintenance and Administration,' and I should be glad if you would kindly correct my markings. I should explain that our advertising account is about £90 in excess of ordinary years, in consequence of advertising the day of the opening of the new buildings, lists of donations, new subscriptions, &c. We never advertise annual subscriptions, only donations. The total of ordinary expenditure was £19,135 7s. 11d."

*** It seems to us that the system of fixing a certain number of beds for occupation out of a larger number of available beds is calculated to lead to confusion without any compensating advantage. After all, the number of beds constantly occupied must ever be the true unit for all calculations. The marked balance-sheet has been returned direct to Mr. Blair, with the information asked for marked thereon.—Ed. *T. H.*

